# Managing the heat: In-Vessel Components

**DOI:** 10.1098/rsta.2023.0408

**Published:** 2024-10-09

**Authors:** Jenny Cane, Alan Barth, Jaime Farrington, Ethan Flynn, Simon Kirk, James Lilburne, Zsolt Vizvary

**Affiliations:** ^1^ United Kingdom Atomic Energy Authority, Culham Campus, Abingdon, Oxfordshire OX14 3DB, UK

**Keywords:** plasma-facing components, STEP, requirements, in-vessel, methodology, heat

## Abstract

The Spherical Tokamak for Energy Production (STEP) programme aims to deliver a first-of-a-kind fusion prototype powerplant (SPP). The SPP plasma places extreme heat, particle and structural loads onto the plasma-facing components (PFCs) of the divertor, limiters and inboard and outboard sections of the first wall. The PFCs must manage the heat and particle loads and wider powerplant requirements relating to safety, net power generation, tritium breeding and plant availability. To enable STEP PFC concepts to be identified that satisfy these wide-ranging requirements, an iterative design (‘Decide & Iterate’) methodology has been used to synchronize a prioritized set of decisions, within the fast-paced, iterative, whole plant concept design schedule. This paper details the ‘Decide and Iterate’ methodology and explains how it has enabled the identification of the SPP PFC concepts. These include innovative PFC solutions such as a helium-cooled discrete and panel limiter design to increase tritium breeding while providing sufficient coverage and enabling individual limiter replacement; the integration of the outboard first wall with the breeding zone to enhance fuel self-sufficiency and power generation; and the use of heavy water (D_2_O) within the inboard first wall and divertor PFCs to increase tritium breeding within the outboard breeding zone.

This article is part of the theme issue ‘Delivering Fusion Energy – The Spherical Tokamak for Energy Production (STEP)’.

## 1. Introduction

The plasma in the Spherical Tokamak for Energy Production (STEP) prototype powerplant (SPP) creates extreme heat, particle and structural loads within the tokamak and creates challenges for the engineering design of the SPP plasma-facing components (PFCs). The viable engineering design space for the SPP PFCs is further constrained by numerous emerging integration challenges related to the spherical architecture and operational needs of the SPP. A novel integrated engineering design approach is required to identify PFC solutions within the context of the wider Vacuum-vessel and In-Vessel Systems (VIVS), tokamak and plant-level functional requirements. The VIVS key functional requirements are to:

— Manage the plasma heat and particle load.
— Achieve fuel self-sufficiency.
— Contribute to net power production (100 MWe minimum).

**Figure 1.**
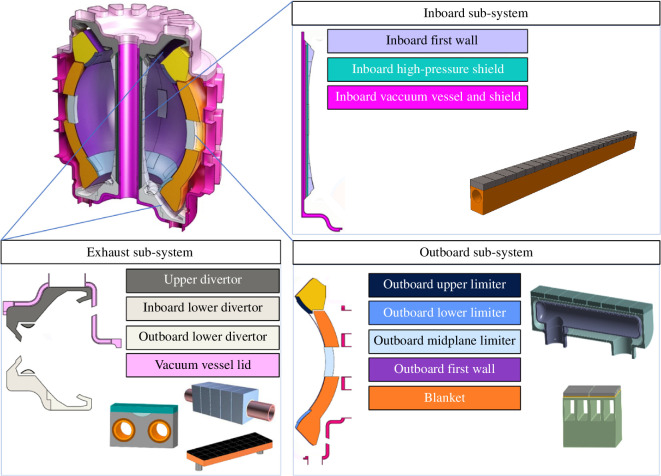
Schematic architecture of the VIVS sub-systems and their constituent components, including the PFCs.

The SPP PFCs are grouped with other components to provide a subset of the VIVS sub-system functionality. VIVS is split into the inboard, outboard and exhaust sub-systems, as shown in figure 1. The sub-systems deliver specific sub-sets of the VIVS functional requirements and the PFCs within each sub-system contribute to these requirements.

## 2. The STEP PFC ‘Decide & Iterate’ methodology

**Figure 2.**
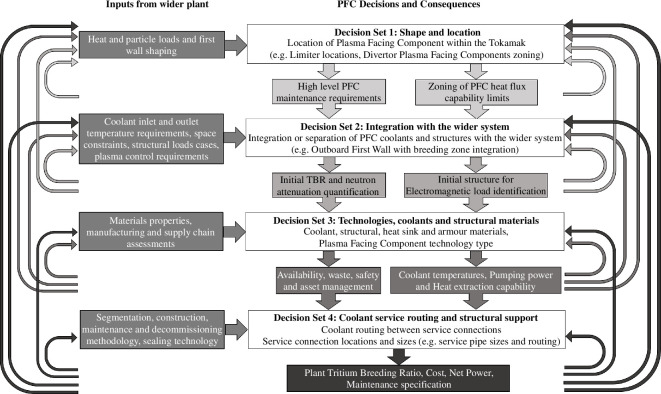
STEP PFC ‘Decide & Iterate’ methodology.

In a traditional ‘top–down’ iterative design approach, the PFC conceptual design would not begin until higher level systems had been well defined. Within a fusion powerplant, however, the design of lower-level sub-systems such as the PFCs has a significant impact on the performance of the fusion powerplant. The ‘Decide & Iterate’ PFC concept design methodology (illustrated in figure 2) has been used for the SPP to provide a practical framework to develop the PFC sub-system designs within the highly constrained and uncertain sub-system, system and plant-level requirement space. Rather than designing the high-level SPP systems before developing the PFC concepts, the ‘Decide & Iterate’ methodology identifies specific aspects of the PFC concept design that have a significant impact on the wider plant and groups them into decision sets. These decision sets are addressed in turn, so that the PFC concept designs are developed from high-level positioning and system integration through component architecture designs to materials, coolants and service routing assessments. By coinciding PFC design decisions with wider plant decisions, the impact of each decision set can be assessed against the sub-system, system and plant-level requirements. This allows for the PFC concepts to be rapidly iterated if it is discovered that they cannot meet their requirements as the fidelity of the plant design system is increased.

The methodology begins with the heat load and wall shaping specifications obtained from the plasma equilibrium and control definitions. The initial heat load specification and first wall (FW) profile is an iterative process itself, which means tools have been developed that allow rapid iteration. This enables ‘Decision Set 1: Shape and location’ to be completed, which defines the PFC wall shaping, heat load zoning and limiter positions. Decision Set 1 coupled with an increased understanding of the plasma control requirements, structural load cases and maintenance strategy enables ‘Decision Set 2: Integration within the wider system’, which identifies how PFCs are integrated with their wider system to enable functional requirements to be met. ‘Decision Set 3: Technologies, coolants, functional and structural materials’ relies on multi-physics analyses, including neutronics, structural, thermal and net power assessments. These design decisions are made in conjunction with materials, manufacturing and supply chain feasibility assessments. At this point in the process, the detail of the PFC designs is increased, enabling plant availability, waste, safety and asset management assessments to be carried out. ‘Decision Set 4: Coolant and service routing’ formalizes the impact of the PFC decisions on service pipework. This important part of the methodology ensures the pipework and structural supports fit within the available space and can be accessed for maintenance. The ‘Decide & Iterate’ methodology enables decision iterations even at ‘Decision Set 4’ if the PFC concept design does not fully comply with the plant fuel self-sufficiency, cost, net power and availability requirements.

The remaining sections of the paper describe how the ‘Decide & Iterate’ methodology was used to determine the SPP PFC concept design and the analysis developed to facilitate this process.

## 3. Heat and particle loads, and FW shaping methodology

**Figure 3.**
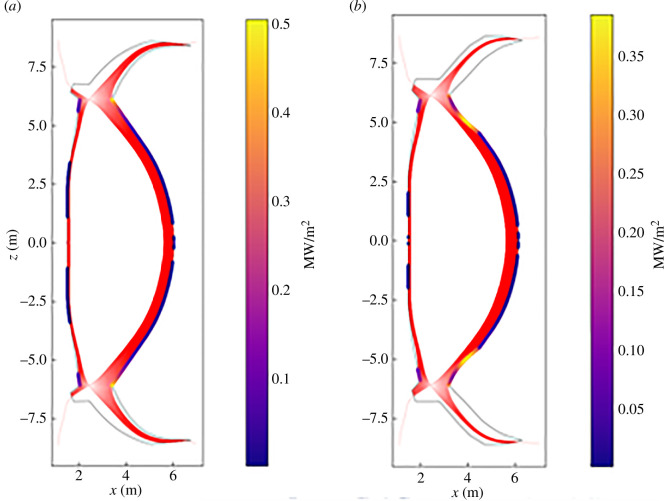
Example of automated adjustment of the FW contour to reduce high heat flux regions of the SPP FW [3]. (*a*) The automatically optimized FW contour. (*b*) The modified contour considering the VDE modelling and incorporating the limiter regions. The plot only shows the main chamber FW-charged particle heat loads.

**Figure 4.**
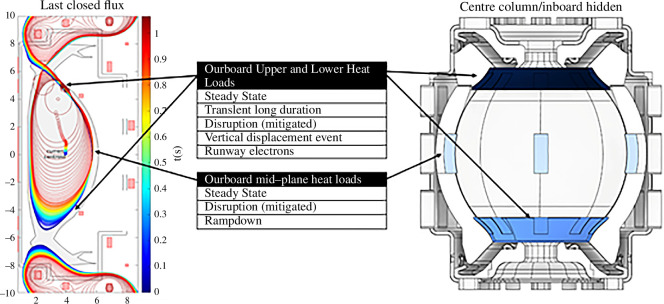
SPP limiter shape, location and heat load types [6].

The heat and particle load and wall shaping requirements for the PFCs are the key inputs to Decision Set 1 of the ‘Decide & Iterate’ methodology. The SPP two-dimensional poloidal FW profile has been integrated into the BLUEMIRA design framework [1,2] with the goal of keeping the steady-state main chamber FW heat loads within defined limits. A summary of the poloidal two-dimensional FW profile design methodology [3,4] is provided below:

— The steady-state flat-top plasma equilibrium is used to generate a preliminary FW contour by assigning initial geometrical features such as plasma last closed flux surface; X-point of plasma; an assumed initial plasma-wall gap; the strike points radial coordinates; divertor apertures and grazing angles.
— This contour enables radiation and two-dimensional thermal charged particle heat loads to be calculated using additional plasma parameters [5]. A bespoke double-null two-dimensional field line tracing code enabling rapid iteration of the contour has been developed to avoid toroidally continuous hot spots caused by steady-state charged particle heat loads (figure 3*a*). This initial FW profile can be generated quickly and provides the initial space claim for the plasma and the required input for transient modelling.
— Transient event modelling is included in the equilibria, and control strategies for these events are developed. Firstly, ‘landing zones’ for vertical displacement events (VDEs) are identified (figures 3*b* and 4, left). This is followed by ramp-up/ramp-down, unplanned high to low (or low to high) confinement (H–L/L–H) transitions and runaway electron (RE) modelling. An initial set of disruption cases (both mitigated and unmitigated) are also identified.
— To calculate the steady-state photonic radiation distribution the CHERAB code [7,8] is used.
— A three-dimensional FW profile, identifying localized hot spots and edge heat loads, is produced and included in the SPP heat and particle load specification. This specification aims to provide surface heat flux values that can be a direct input to the engineering analysis of the components [9]. To fully populate the specification, further development of the heat and particle load analysis is required. This includes three-dimensionally charged particle tracing (e.g. SMARDDA [10]), volumetric heating owing to neutrons (MCNP [11] and OpenMC [12]) and fast particle (alpha) modelling (LOCUST [13]) considering the ripple field and other perturbations (resonant magnetic perturbation, error field correction coils, etc.). It is understood that with the increasing maturity of the design, the heat loads must be kept updated to consider the segmentation of components, gaps, engineering tolerances, etc. The results of these more detailed analyses will also feed into the detailed three-dimensional surface design of the PFCs.

The SPP divertor wall shape has been defined with the aim of achieving access to a double-null, detached plasma regime, to reduce heat and particle loads on the PFCs and allow for sufficient pumping to minimize the build-up of impurities (e.g. helium) within the divertor [5]. Engineering constraints, such as allowing space for shielding materials and coolant pipes, are considered within Decision Set 4 of the Decide & Iterate methodology, to arrive at a final configuration. The current SPP heat and particle load specification has established that during steady-state conditions, the expected FW heat loads are below 1.3 MW/m^2^ and 5–10 MW/m^2^ at the divertor strike points. It is intended that the load specification will also cover the expected PFC erosion rates. Preliminary studies have been conducted for an earlier SPP concept [14] (using ERO [15] and ERO 2.0 [16]), but further work is needed for the latest iteration.

Planned transient events such as plasma ramp-up and ramp-down result in localized high heat loads both on the FW and the divertor lasting tens of seconds. Unplanned transient events such as VDEs, RE, unmitigated and mitigated disruptions, high to low (or low to high) confinement (H–L/L–H) transitions [17–22] and Edge Localized Modes (ELMs) [23,24] result in extreme heat loads on the PFCs lasting from a few to tens of milliseconds and estimated in the range from hundreds of MW/m^2^ to hundreds of GW/m^2^ [25] (similar to EU-DEMO [26]). Ultra-High Heat Flux (UHHF) limiter components need, therefore, to be included at specific locations to mitigate the effects of these extreme heat loads on the VIVS and wider plant.

### (a) Decision Set 1: shape and location

#### (i) Limiters placement and coverage

The placement and coverage of the limiters provides a case study for Decision Set 1 in the ‘Decide & Iterate’ methodology. Limiters must be designed to allow sufficient neutron flux through to the breeding zone to limit their impact on global reactor TBR, since fuel self-sufficiency demonstration is a primary objective of STEP [27]. This limits the material volume, choice of material and coolant. A loss of coolant accident (LOCA) from UHHF transient events represents a substantial safety and asset protection hazard, so it must be mitigated as far as reasonably practicable [28]. Hence, the SPP wall protection strategy uses limiter PFCs with enhanced heat flux capability compared to the majority of the outboard first wall (OFW). These limiter PFCs are strategically placed at the locations identified in the heat and particle load specification (figure 4).

The SPP concept includes outboard upper and lower limiters with full toroidal coverage to protect the rest of the OFW from plasma contact during both symmetric and asymmetric unmitigated VDE and RE events. Although these events are expected to occur infrequently, it is anticipated that they will cause considerable armour melting. The SPP concept therefore enables sections of the upper and lower outboard limiters to be replaced without removing the SPP vacuum-vessel lid. This is achieved by designing the limiters to have horizontal port-removable ‘discrete’ sections that are biased to suffer damage during VDE and RE events, located between vertically removable ‘panel’ sections that are recessed compared to the discrete sections. Four outboard midplane discrete limiters are included to protect the OFW during plasma ramp-down and any unplanned outwards radial plasma movement. Mitigated disruption events are expected and will result in a radiative heat load across the limiters, OFW and divertor.

Horizontal port-based maintenance of an inboard limiter is not practical within the SPP concept as this would require complex maintenance equipment in a harsh radiation environment, re-joining of irradiated pipes and allocation of valuable inboard radial space for removable features. Therefore, the increased heat handling functionality for plasma ramp-up is to be achieved by the Inboard First Wall (IFW) instead (essentially, a wall limiter). The IFW is maintained vertically after the removal of the SPP vacuum-vessel lid, so H–L transition plasma events that result in a full-energy plasma impacting the IFW should be minimized to avoid the need for unplanned vertical maintenance to replace damaged components, as this would be detrimental to plant availability. A radial space allocation for an inboard plasma-wall gap has been included to provide a margin for the plasma to be controlled during an H–L transition event. The intention is to reduce the likelihood of a full-energy plasma impacting the IFW to the extent that this only occurs during fault conditions, such as when plasma control may be significantly compromised owing to a fault in the confinement system. If achieved, then confidence in the serviceable lifetime of the IFW is increased, instances of unplanned maintenance for the IFW are reduced and the overall plant availability is increased. As the overall SPP design maturity of supporting systems, such as diagnostics, is developed, the plasma-wall gap space allocation requirements will be re-assessed. Any inboard plasma-wall gap comes at the expense of valuable inboard radial space that could otherwise be used for shielding the magnets. Therefore, if further, more refined analysis indicates that the frequency of adverse H–L transition events is unacceptable with the current inboard plasma-wall gap, or that a discrete inboard limiter is required in that space, then the ‘Decide & Iterate’ methodology will be used to allow the design to be modified with due consideration to inboard shielding performance.

#### (ii) Divertor target placement

**Figure 5.**
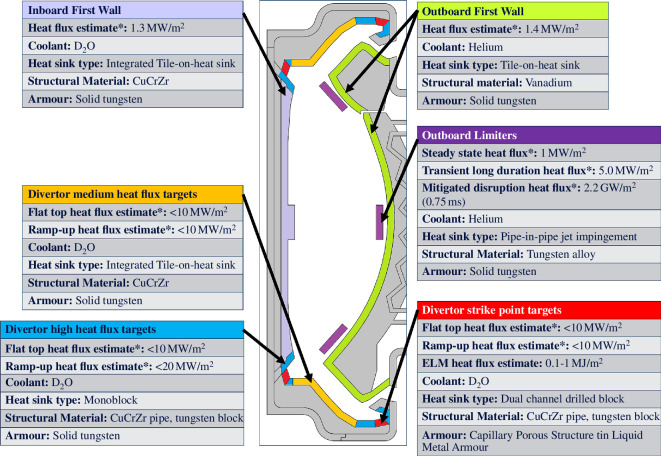
Summary of the PFC designs selected to meet the varying functional requirements of the SPP concept in-vessel systems. This shows the estimated (asterisk indicates peak steady state) heat flux, the coolant, heat sink, structural material and armour for each component.

A similar zoning/placement activity has been undertaken on the divertor. By considering the requirements of the PFCs in different areas of the SPP concept design, it is possible to split the divertor into zones with different heat and particle load requirements (figure 5). This enables high-complexity, monoblock PFC designs to only be used within the regions where high heat load capability is required. For the remaining areas of the divertor larger and less complex tile-on-heat sink PFCs are specified, providing a route to increasing the exhaust sub-system reliability owing to the reduced total part count.

As set out in Vaccaro *et al.* [3] and Romanazanov *et al*. [16], the SPP concept plasma exhaust solution is a detached, double-null configuration. Based on a ‘perfect’ double-null scenario, the methodology described in Decision Set 1 estimates that away from the strike points the flat-top surface heat flux on the divertor PFCs will be <5 MW/m^2^. Margin has, however, been included in the divertor PFC designs to account for asymmetries in power sharing between the upper and lower divertors as well as increased surface heat flux owing to the three-dimensional shaping of PFCs. Areas located away from the strike points are, therefore, designed to withstand a maximum heat load of 10 MW/m^2^.

Although the SPP plasma scenario will be designed to achieve ELM-free operation [5] and mitigation systems will be implemented for ELM control [25], the SPP concept still requires the regions in the vicinity of the divertor strike points to withstand 10–20 MW/m^2^ for short periods of time. These loads could be caused either by a temporary loss of detachment during the final phases of plasma ramp-up or owing to the effect of vaporization in the adjacent strike-point PFCs during unmitigated transient events such as ELMs [29].

### (b) Decision Set 2: integration with the wider system

#### (i) OFW integration with breeding zone

The design of the SPP OFW provides an example for Decision Set 2 within the STEP PFC ‘Decide & Iterate’ methodology. The OFW forms part of the outboard sub-system, for which the primary functional requirements are to breed and deliver sufficient tritium to achieve fuel self-sufficiency [27] and to deliver high heat-grade coolant at 600°C [30]. The OFW must contribute to this while safely achieving a range of demanding requirements, so understanding how each OFW decision impacts the overall performance of the outboard sub-system is key.

An example of this requirements trade is the decision to structurally integrate the OFW with the breeding zone, including the integration of the coolant system. This integration increases the TBR and high-grade heat compliance. The supply of coolant in series from the OFW to the breeding zone enables a larger proportion of thermal energy to the primary power cycle in the form of high-grade heat. The OFW forms the front and side skin of the pressure boundary for the breeding zone, which reduces the total structural material between the breeding zone and the plasma, thereby increasing the achievable TBR by approximately 5% [31]. This integration does, however, require an integrated design approach for the OFW and breeding zone coolant cycle to ensure adequate heat removal through the OFW (primarily surface heating) and the breeding zone (primarily volumetric heating), while ensuring an outlet of 600°C.

#### (ii) Divertor outboard leg integration with outboard sub-system

The Decision Set 2 methodology has also influenced the integration of the OFW with the divertor PFCs. The similarity of the heat capability requirements of the ‘low heat flux’ PFC region of the divertor and OFW has led to the consolidation of this region of the divertor into the wider outboard sub-system, as shown in figures 1 and 5. This reduces the structural material in front of the breeding zone behind the divertor and facilitates the extraction of the extended breeding zone during maintenance.

#### (iii) Separation of divertor PFC and support structures

**Figure 6.**
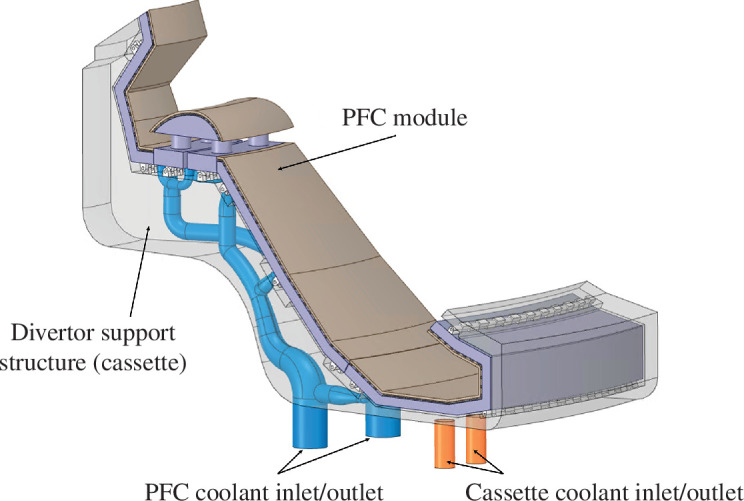
SPP concept design showing separated divertor PFC and divertor support structures.

Decision Set 2 within the ‘Decide & Iterate’ methodology can also facilitate decisions to separate components within systems that have typically been consolidated in other tokamak designs. An example of this is the separation of the divertor PFC module from its support structure (cassette), as shown in figure 6. This separation allows the PFC and cassette cooling loops to be optimized for their respective heat loads and pumping requirements [32]. It also provides the potential opportunity to replace the shorter-lifetime PFCs without replacing the longer-lifetime cassettes.

### (c) Decision Set 3: technologies, coolants and materials

Following Decisions Sets 1 and 2, where shape, location and integration requirements are defined, technology decisions can be made for the PFCs. These decisions involve numerous requirement trades that embody the integrated nature of the ‘Decide & Iterate’ methodology. This section presents the key requirement trades and technology, coolant and material options available during the SPP concept design process before presenting the decisions made for the SPP concept. Throughout this process, the impact of neutrons must be considered, both in terms of material degradation and volumetric heat loads. Material damage and material property degradation are not well known for most PFC materials at the diaplcements per atom (dpa) levels expected when the SPP is producing power and tritium. At this stage of the SPP concept design, it is, therefore, necessary to carry out parameter sensitivity studies and include these uncertainties in the analysis and component assessments.

Coolant selection is driven by the ability to handle the required thermal loads, both in terms of providing suitable heat transfer to maintain PFC materials within their acceptable operating temperature range and allowing for acceptable temperature increases across the coolant system. This must be balanced against other factors, such as minimizing pumping power, handling neutrons appropriately, and must account for the material degradation attributed to neutrons, in addition to erosion of the PFC armour due to plasma interactions. Water enables low pumping power, good heat flux handling and provides excellent neutron shielding capability. Similarly, selecting heavy water (D_2_O) rather than light water (H_2_O) within the non-breeding (inboard and exhaust) sub-systems encourages neutron scattering to aid tritium breeding within the Outboard sub-system. Gas coolants generally have poorer heat flux handling and require higher pumping power than water, but are more neutron transparent, which aids tritium breeding if they are used within the OFW PFCs at the cost of shielding performance. Additionally, safety must be considered at all stages, including in the event of LOCAs, where mitigation sub-system requirements are expected to be more stringent for water than for gases. Therefore, gases are preferable as coolants for UHHF PFCs if the PFC heat handling requirements can be achieved.

To minimize cost and complexity, it is preferable to consolidate the coolants used within the VIVS system, and if a top-down design methodology was used, the restriction on coolants would be a likely additional constraint to PFC design. During the design of the SPP, however, it has become clear that the functional performance of the PFCs cannot be met using a single coolant type. The ‘Decide & Iterate’ methodology provides the flexibility to identify coolants that not only enable the PFC requirements to be met but also contribute positively to the plant-level requirements. The reasoning for the PFC coolant selection for each sub-system is summarized in the sections below, illustrating how these low-level sub-system concept design decisions have significant impact on the higher level system performance.

Armour materials must have sufficient thermal and structural properties to withstand thermal loads and conduct heat efficiently to the coolant, while also being resistant to erosion when impacted directly by plasma particles. Preliminary erosion rate [14] and neutronic calculations [11,12] coupled with sensitivity analyses have enabled appropriate thicknesses of solid armour to be identified for solid tungsten that provide sufficient lifetime and neutron transparency to meet the SPP PFC requirements. Studies on existing tungsten wall tokamaks like WEST, a world leader in this field, have provided valuable data on erosion rates and impurity production [33,34], and recent advancements from DIII-D’s research on tungsten armour tiles have significantly enhanced understanding of the mechanisms responsible for increased erosion [35]. Additionally, progress in validating models of tungsten erosion, re-deposition and migration [36] will be crucial for optimizing the design and operation of future devices like STEP.

Liquid metal armour (LMA), particularly lithium and tin, as an armour material takes advantage of the vapour shielding phenomenon to reduce incident heat flux on the underlying material, increasing the ability to handle off-normal transient events. LMA PFCs have been shown to survive heat loads comparable to the SPP ELMs [37,38], although experimental set-ups were not fully representative of a tokamak environment and further development is required. LMA does, however, come with challenges such as complex integration and the need to minimize the transport of materials with high atomic proton numbers into the core plasma.

Heat sink material choice is driven by thermal and structural properties, compatibility with the coolant and acceptable operating temperature ranges. Neutron irradiation severely damages materials and leads to a loss in thermal conductivity, activation and embrittlement. Eurofer97, a reduced-activation ferritic martensitic steel with desirable properties such as high strength and low activation levels when irradiated serves as a good example of the impact neutron irradiation has on material selection. When operated below 350°C, Eurofer97 loses almost all ductility at very low levels of neutron damage [39] (<0.1 dpa, equivalent to a few days of SPP deuterium–tritium operation) making it unsuitable for use in thin-walled pipes owing to the unacceptable risk of failure. This renders Eurofer97 incompatible with water unless it is operated above 350°C, which would significantly reduce the margin to critical heat flux, saturation temperature or require the SPP PFCs to operate with supercritical water conditions. A typical alternative [40–42] is copper chromium zirconium (CuCrZr), a copper alloy that combines excellent thermal properties with good structural properties. It has a useable temperature range of between 200°C, limited by embrittlement [43,44], and 350°C, limited by creep, over-ageing and irradiation-assisted corrosion [45]. Its high thermal conductivity also allows for thicker sections and thus offers increased design flexibility. Data exist [43,44] to suggest that CuCrZr retains acceptable properties up to 10 dpa, although development is needed to better understand irradiated performance. It is therefore seen as the only viable option for water-cooled designs. Neither Eurofer97 [39] nor CuCrZr is considered to be suitable for temperatures above 550°C, so operating at elevated temperatures requires the use of more novel materials such as tungsten alloys.

The PFC heat sink technology type is also chosen during Decision Set 3. Firstly, the coolant channel design must be selected. Jet impingement configurations offer a route to enhancing heat handling capability for gas-cooled components but come at a cost of added complexity. The optimal configuration is, therefore, closely linked to coolant choice. Gas cooling for high heat flux applications typically requires jet impingement PFCs to achieve the required heat transfer, while water, owing to its superior thermal properties, can achieve sufficient heat transfer with simpler cooling channel designs. The design of the heat sink and armour must then be defined. Tiled armour bonded to an underlying heat sink offers benefits in neutron transparency owing to a reduction in tungsten volume and is compatible with large or complex-shaped heat sinks. However, the risk of armour delamination limits heat flux handling ability [46–48], so ‘tile-on-heat-sink’ arrangements are best suited to lower heat flux regions. Monoblock PFCs have been developed extensively within the fusion community [48–50] to handle heat fluxes up to approximately 20 MW/m^2^ and address delamination risks. Monoblocks are generally preferred in higher heat flux regions, but their size is limited, leading to high part counts, and the configuration requires simpler heat sinks such as cooling pipes.

#### (i) OFW coolant

For the OFW at Decision Set 3, helium coolant with a tile-on-heat sink design has been selected and integrated into the skin of the breeding zone. The proximity of the OFW to the liquid lithium breeder and the decision to place the OFW in series with the breeding zone drive the need to use helium as it provides an (As Low As Reasonably Practicable) ALARP safety and asset protection system for the SPP concept. The neutron transparency of helium also maximizes TBR and consolidates the OFW coolant with the breeding zone primary coolant [27].

#### (ii) Limiters coolant, heat sink technology and material

**Figure 7.**
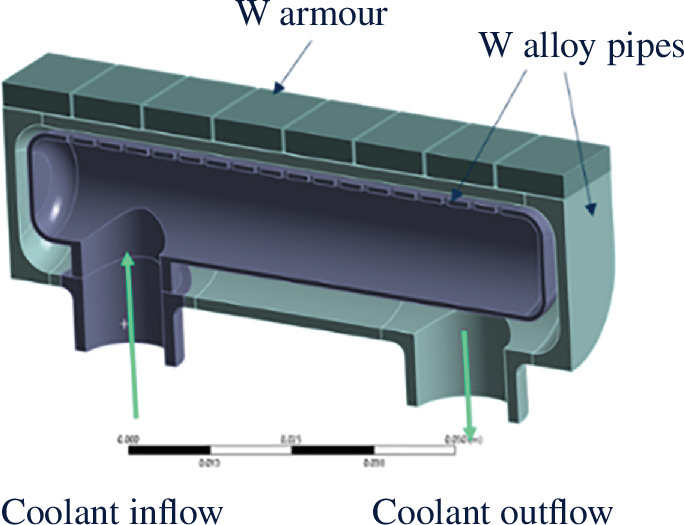
Limiter helium-cooled jet impingement PFC.

Owing to the sacrificial nature of limiters, it is crucial that the impact of a LOCA event is minimized. Helium is, therefore, the preferred coolant compared to water both to minimize the rate of pressure increase in the plasma chamber and to reduce the impact of any breeder/coolant interactions. Within Decision Set 3, it was identified that helium-cooled limiters could be designed to meet the SPP expected transient event heat flux and pumping power requirements if a pipe-in-pipe jet impingement PFC type is selected (figure 7).

This arrangement requires the use of a structural material that can be operated from approximately 400°C up to 600°C at the armour interface during steady-state operations. A tungsten heavy alloy has been identified as the most credible heat sink material option to maximize the thermal handling performance during transient events. Specific compositions of this material show increased ductility when compared to traditional tungsten across the required operating temperature range [51]. Jet impingement concepts using tungsten alloy have been developed for EU-DEMO [52]; however, it is acknowledged that significant risk remains around the use of tungsten heavy alloys including uncertainty around the ductility of tungsten alloy at the assumed coolant temperatures and irradiation levels. STEP plans to further assess the suitability of tungsten alloy in relation to the specific limiter requirements.

#### (iii) Inboard First Wall (IFW) PFC coolant and heat sink material

Although the primary function of the IFW is to protect the inboard sub-system components from the average flat-top and ramp-up heat loads (approx. 1.3 MW/m^2^), the IFW must also contribute to shielding the centre column magnets [53], even if just by minimizing the radial space required to achieve the heat load management functionality. A top-down design methodology would be likely to constrain the IFW coolant to helium to be consistent with the OFW. However, to reduce the thickness of the inboard sufficiently to meet the SPP size requirements, the ‘Decide & Iterate’ methodology was required. By focusing on the requirements of the sub-systems in parallel to the high-level system requirements, this methodology provided the flexibility to identify that the inboard shielding should use water as a coolant, owing to its superior neutron shielding properties compared to helium. With water-cooled inboard shielding provisionally decided, changing the IFW coolant from helium to water can further results in a combined improvement in the overall shielding of the centre column magnets of around 5–10% [54]. The importance of low-level sub-system design decisions on plant-level requirements is further shown when the impact of the IFW coolant on the powerplant TBR is considered. The increased neutron scattering of D_2_O compared to H_2_O results in a greater number of neutrons reaching the breeding zone, and a TBR increase of 0.015 [55] was calculated, benefiting SPP fuel self-sufficiency. Therefore, although H_2_O would have offered marginally better heat extraction and neutron shielding properties, the confidence in SPP fuel self-sufficiency was prioritized and D_2_O was selected over H_2_O as the IFW coolant.

**Figure 8.**
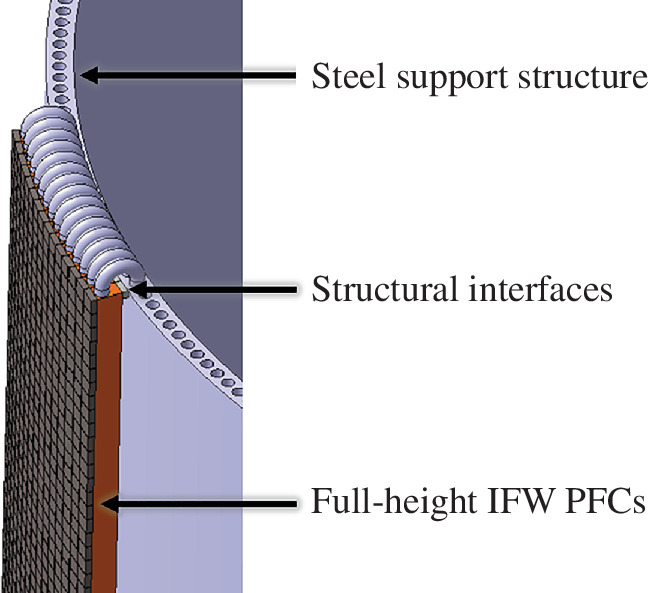
SPP IFW concept illustrating full-height tile-on-heat sink PFC design.

The selection of a D_2_O coolant for the IFW provided opportunities to select CuCrZr as a heat sink material. In particular, the significant overlap in acceptable temperature ranges of water and CuCrZr may allow for a larger coolant temperature rise in the IFW, which is helped further by the excellent thermal conductivity of CuCrZr moderating heat sink temperatures. A larger temperature rise is not only good for power generation, but it also provides opportunities to have longer channels in the PFCs of the IFW, reducing the number of pipe connections and bends. This opportunity is embodied by the SPP concept design for the IFW comprising ‘full-height’ tile-on-heat sink PFCs that run from the lower to the upper end of the IFW region and back through a support structure, as illustrated in figure 8.

Reducing the number of pipe connections and bends not only reduces complexity but could also help to minimize inboard radial space occupancy of the IFW. There are, however, integration challenges related to the selection of CuCrZr for the IFW heat sink. The high electrical conductivity of CuCrZr means the shape, size and positioning of the IFW needs to be carefully designed to minimize electromagnetic loads and to ensure stability of the plasma during operations. As a partial mitigation, a low irradiation swelling steel with significantly lower electrical conductivity than CuCrZr has been selected for use in the support structure that holds the ‘full-height’ IFW PFCs through structural interfaces, as illustrated in figure 8. Further design iterations, analysis and material data will aim to realize opportunities and address emerging integration challenges.

#### (iv) Divertor coolant, heat sink technology and material

The ‘zoned’ PFC target approach decided within Decision Set 2 enables three different types of PFC to be proposed for the SPP concept design, as shown in figure 5. D_2_O coolant is used for all divertor PFCs as it provides excellent heat flux handling, low pumping power, good shielding performance and tritium breeding benefits. The use of D_2_O within the IFW and divertor PFCs does increase the impact of a LOCA, compared to a helium coolant particularly if lithium is also present within the plasma. As outlined in (iii) above, the Decide & Iterate methodology does, however, identify that the SPP functional performance requirements—particularly related to lifetime, fuel self-sufficiency and tokamak size—can only be achieved if a water-based coolant is used. The design options for safety systems, such as a vacuum-vessel pressure relief, will therefore need to be considered carefully, specifically in terms of overpressures owing to steam and/or helium expansion and the potential for lithium and/or corrosive reaction products to affect the functionality of the system [28].

The lower thermal stress in the medium heat flux regions enables tile-on-heat sink PFCs to be selected. This reduces part count, the number of welds and complexity when compared to monoblock alternatives (where typically large numbers of relatively small monoblocks are required to cover the heat flux region). Monoblock or ‘drilled-blocks’ designs are restricted to the areas subjected to heat loads above 10 MW/m^2^ (figure 5) [50,56,57]. The monoblocks use heat transfer enhancing features such as internal swirl tapes to maximize heat flux handling capability.

Capillary Porous Structure (CPS) tin LMA has been selected for the strike point regions of the divertor to provide an additional mitigation if the plasma scenario cannot be made completely ELM free. The LMA PFC design is expected to be more resilient to transient loads, particularly ELMs owing to the enhanced vapour shielding effect provided by the liquid tin when subjected to heat fluxes in excess of 20 MW/m^2^ [37,58]. The tungsten CPS required to draw and hold the liquid tin in place does not have a structural role and can therefore sustain more damage, such as severe recrystallization, without affecting the performance of the PFCs. The small pore sizes within the porous layer (approx. 100 µm) provide capillary pressure to drive tin to the surface to replenish any material lost via evaporation, sputtering or other means. The small surface feature size helps to mitigate against droplet ejection caused by surface instabilities owing to currents induced in the surface layer.

The use of tin as the liquid metal armour has been selected to reduce the safety implications of a LOCA event where the water coolant within the LMA PFC mixes with the LMA. The safety implications of tin and water interactions have been assessed, and although it is anticipated that a protection system will be needed, it has also been highlighted that the impact of a tin/water interaction will be lower than a lithium/water interaction event. The major limit placed on LMA is the rate of tin evaporation, especially during normal operations, and the impact that this has on the plasma performance. Furthermore, excessive amounts of tin migrating towards the exhaust pumping system could also be problematic. The ‘zoned’ PFC target approach is, therefore, an important feature of the SPP design as this enables the total surface area covered with LMA to be kept to a minimum.

### (d) Decision Set 4: coolant service routing and structural support

The final decision set of the PFC ‘Decide & Iterate’ methodology is highly dependent on the decisions made in the previous three decision sets. This is because the coolant types, approximate mass flows and pressures must be decided to be able to assess the service routing and structural support requirements. The successful completion of Decision Set 4 is, however, critical to ensure that the PFC concept designs comply with wider system and plant-level requirements.

An example of this dependency is that Decision Set 3 has identified the need to use different coolants to meet the requirements of the SPP PFCs and that a tin liquid metal armour loop is required to provide additional mitigation against ELMs within the divertor. This increases the complexity of the in-vessel systems and the service routing to them, which is likely to reduce the availability and maintainability of the SPP concept. Within Decision Set 4, a decision must be made whether the use of several coolant types is justified to meet the high-level requirements of STEP or if a lower complexity device with lower performance is preferable.

Decision Set 4 has also highlighted that the routing of IFW and divertor D_2_O coolant service pipes through the base of the tokamak [59] is highly restricted and presents integration challenges. The proximity of the divertor inboard leg, converging toroidal field coils and position of the poloidal field coil and vacuum-vessel support structures force the inboard coolant service pipes radially outboard before penetrating the vacuum-vessel, ultimately leading to a larger inboard sub-system, which increases access and storage space requirements.

Similarly, there is currently insufficient space for the OFW and breeding zone 80 bar helium coolant service pipes within the SPP concept upper and lower radial ports. There is also concern that suitable sealing technologies for the 80 bar, 600°C helium may not be possible. At this point in the design cycle, it is, therefore, necessary to reassess previous design choices and identify an adjusted design point for the SPP concept.

## 4. Conclusions

The ability to manage the SPP concept heat and particle loads is provided by discrete and panel limiters, OFW, IFW and divertor PFCs. The conceptual designs of the PFCs have been developed through a ‘Decide & Iterate’ methodology process that ensures the consideration of key plant-level requirements when PFC locations, segmentation, materials, technology types and service routing are selected.

The initial inputs into the PFC ‘Decide & Iterate’ methodology are the plasma equilibrium and heat and particle loads, which define the FW and exhaust shapes. Tool development in this area has been crucial to speed up the development of the SPP concept with advances in the automation of wall shaping to allow a quick generation of the initial FW. The SPP heat and particle specification provides the information required to determine the location and size of the limiters and bounds the heat extraction requirements of the inboard and outboard FWs. It has also provided the opportunity to introduce a zoned PFC approach in the divertor, which reduces the part count and complexity of the divertor using tile-on-heat-sink over the majority of the divertor surfaces, with higher complexity monoblock and liquid metal armour designs reserved for high heat flux and strike point regions.

The PFC ‘Decide & Iterate’ methodology helps identify where sub-systems should be integrated and separated to enable systems to meet their functional and performance requirements. Within the SPP outboard sub-system, this has resulted in the decision to join the OFW structurally and thermally with the breeding zone. This improves the critical plant-level requirement to achieve fuel self-sufficiency and contributes to the ability to deliver 600°C high heat-grade coolant to the SPP power cycle. For the SPP exhaust sub-system, this section of the process has resulted in the decision to separate the divertor PFCs and structural support. This enables the system to achieve the divertor heat extraction requirements while keeping pumping power to acceptable levels. The PFC ‘Decide & Iterate’ methodology has also facilitated the decision to integrate the similar heat flux regions of the divertor outboard leg upper surface into the OFW, which improves tritium breeding and maintenance of the outboard sub-system. For the inboard sub-system, the increased heat handling functionality for plasma ramp-up is to be achieved by the IFW rather than a dedicated discrete limiter.

With regard to selecting coolants, structural materials and technology types, the PFC ‘Decide & Iterate’ methodology provides a mechanism to manage the design iterations needed to balance the wide-ranging and highly challenging PFC heat extraction, tritium breeding, net power, maintenance, availability, waste, lifetime and cost requirements. Within the STEP programme, this has resulted in solutions that are likely to be applicable to other fusion machines, such as the use of panel and discrete limiters, full-height IFW PFCs, and the zoned use of liquid metal armour to mitigate ELMs.

The final stage of the ‘Decide & Iterate’ methodology has highlighted several issues relating to the complexity of the service routing and difficulties fitting coolant pipework through ports. This is recognized as an expected outcome at this point in the concept design of a first-of-a-kind prototype powerplant, and the PFC ‘Decide & Iterate’ methodology will provide an efficient way of mapping the design space and iterating the SPP PFC concept. By continuing to follow this methodology and using and expanding the tools and capabilities of the STEP team, there is confidence that an adjusted set of PFC solutions will be developed that will both manage the SPP heat and particle loads, in addition to enabling the wider STEP plant-level requirements to be successfully achieved.

## Data Availability

Accessible data will be shared via UKAEA Open Data Register.
